# Ensuring the spread of referral marketing campaigns: a quantitative treatment

**DOI:** 10.1038/s41598-020-67895-6

**Published:** 2020-07-06

**Authors:** Sayantari Ghosh, Kumar Gaurav, Saumik Bhattacharya, Yatindra Nath Singh

**Affiliations:** 1 National Institute of Technology Durgapur, Durgapur, India; 2 Indian Institute of Technology Kanpur, Kanpur, India; 3 Indian Institute of Technology Kharagpur, Kharagpur, India

**Keywords:** Applied mathematics, Complex networks

## Abstract

In marketing world, social media is playing a crucial role nowadays. One of the most recent strategies that exploit social contacts for the purpose of marketing, is referral marketing, where a person shares information related to a particular product among his/her social contacts. When this spreading of marketing information goes viral, the diffusion process looks like an epidemic spread. In this work, we perform a systematic study with a goal to device a methodology for using the huge amount of survey data available to understand customer behaviour from a more mathematical and quantitative perspective. We perform an unsupervised natural language processing and hierarchical clustering based analysis of the responses of a recent survey focused on referral marketing to correlate the customers’ psychology with transitional dynamics, and investigate some major determinants that regulate the diffusion of a campaign. In addition to natural language processing for topic modeling, detailed differential equation based analysis and graph theoretical treatment have been carried out to explore the conditions of success for the campaign in terms of realistic parameters both for homogeneous and heterogeneous population structure. Finally, experiments have been performed for generation of a recommendation network to understand the diffusion dynamics in realistic scenario. A complete mathematical treatment with analysis over real social networks helped us to determine key customer motivations and their impacts on a marketing strategy, which are important to ensure an effective spread of a designed marketing campaign. Because of its systematic generalized formulation, the prescribed quantitative framework may be useful in all areas of social dynamics, beyond the field of marketing.

## Introduction

Online social networks have become an undeniable accessory in today’s life. People habitually use online social networks for conveying information as well as opinion due to the convenience, competence, and substantial dissemination power. Use of social networks is increasing day by day among political campaigners and marketing managers for promoting an idea, a product or a brand. If a marketer encourages consumers to share and spread a marketing message through their social contacts, it is called Referral Marketing. As the spread of the message can have an epidemic-like effect, this is also commonly termed as Viral Marketing (VM). Over the past decade, the domain of VM has grown explosively, which now includes passing along advertisements, photos, videos, promotional hyperlinks, animations, games, newsletters, press releases etc. to promote a particular product. In last few years, several studies^1–4^ started to conceptualize VM as a close derivative of disease infection models from mathematical epidemiology. It has been pointed out that understanding the contagion in a population from the perception of a mathematical epidemiologist will be considerably beneficial for the marketers for planning VM campaigns in a more organized and methodical manner.

Model formulation, study and analysis of epidemics have been applied to several problems beyond the boundaries of health and biology over past decade, to successfully depict and understand these phenomena, and device strategies. Starting from the theoretical papers by Kermack and McKendrinck^5^, infectious disease models have been vastly applied to analyse the spread of information, rumor, custom, scientific ideas, opinions, petitions etc^6–9^. In the classical models of epidemiology, the spread of infectious diseases depends mainly on the interactions between susceptible and infected, while the dynamics becomes more complex and nonlinear as the models describe more realistic social scenarios. An epidemic, typically, is defined as a situation in which the number of the infected reaches a significant percentage at steady state. In the case of a VM campaign, it would be a situation where the sharing via social network creates enough momentum so that the marketing message reaches and attracts a majority of its target consumers. In this particular context, purpose of the study will be focused on maximizing the spread, while the usual epidemiology studies aim to contain the epidemic, which in itself adds an interesting perspective to the problem. The spread of marketing messages in social networks raises various theoretical and practical questions: How can an advertisement reach maximum audience? Beyond the design and content, are there any factors that affect this dynamics? What actions can the companies take to speed up the diffusion rate?

In marketing, Bass^10^ used underlying epidemic model as a foundation for his new product diffusion model. But, in the context of online social networks and digital contagion, Sohn et al.^11^ first demonstrated the VM diffusion as SIR and SEIAR processes of epidemiology in very recent times. Rodrigues et al.^12^ have proposed a mathematical model of the VM progression, using insights from epidemiology with quantitative treatment. Bhattacharya et al.^13^ demonstrated a more realistic model of VM propagation, where some essential feedback interactions were considered for the first time. However, these models^11–13^ do not reflect the spreading process adequately. Though there are several survey-based studies and marketing data available for VM diffusion which clearly indicate the complex and nonlinear interaction in a population, most of these studies stick to the classical epidemiology models to depict and understand the scenario. An extensive survey by Ghosh et al.^14^ has revealed that beyond the viral components from creative perspective, a clear insight of customer behaviour becomes indispensable for ensuring the relevance and survival of a newly-launched campaign. With the goal to develop a model which is more realistic and data-driven, they implemented the major observations of the survey to create a conceptual framework for diffusion of VM advertisement in society.

In our present study, we analyze the data collected from recent surveys^14–17^, focusing mostly on qualitative studies^18^, which gives a room for introduction of new and complex interactions, as well as conceptual frameworks. First, using Natural Language Processing (NLP) tools, we figure out several important dynamical aspects which need to be incorporated into the model to describe the dynamics more appropriately. As unsupervised algorithms can find out the important as well as latent structures in a dataset, which are difficult to visualize otherwise, we use hierarchical clustering and Latent Dirichlet allocation (LDA) to analyze the survey responses. Next, we propose a flow chart of the dynamics based on causation analysis, which aligns much better with reality and develop a holistic model for epidemic-like spreading of VM campaigns. To understand the VM dynamics, mean-field equations are derived and numerical simulations are carried out. The dynamics have also been studied keeping heterogeneity of social systems in mind and extensive simulation based studies were carried out over random, scale-free and real social networks. Due to the unavailability of an existing recommendation network structure in public domain, an experiment has been carried out to generate the structure of a recommendation network and to study the dynamics on recommendation-based contact network. Our overall study provides a prescription to derive a mathematical model purely from extensive analysis of survey data, and to further examine it for better understanding and predictability of social contagions.

**Figure 1:**
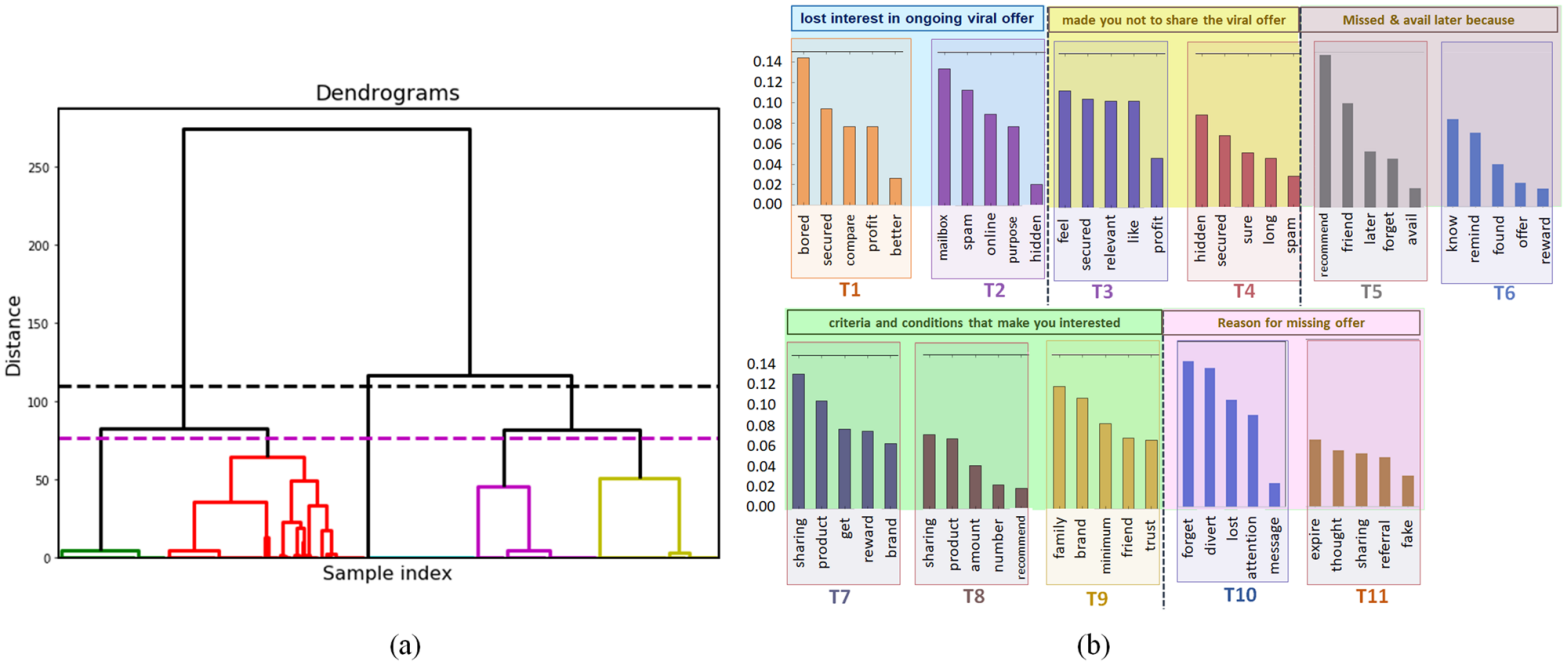
Analyses of survey data: (**a**) Hierarchical clustering of polar responses to detect the sub-population as described in “Identification of subpopulations from polar questions”. Thresholding at the black horizontal line gives the main three sub-populations as shown Fig. 3, and threshloding at the magenta horizontal line gives the people category as defined in Table 1. (**b**) Topic modeling of processed response. Some selected topics (T1–T11) from different open ended questions are shown here. For better visualization, we show five words with highest scores in each topic.

## Quantitative and qualitative analyses of survey data

### Identification of subpopulations from polar questions

To understand the driving factors of VM dynamics from consumer psychology perspective, we extensively analyze the collected survey results of Ghosh et al.^14^. In most of the previous studies, the consideration of SIR process for studying VM diffusion and the assumption of Susceptible, Infected and Recovered subpopulations in the society are based of speculative arguments^11,12^. Considering this to be the staring point, we attempt to draw conclusions about the model structure from data by analysing eight polar questions of the mentioned survey^14^. This questions have predefined options (e.g., ‘Yes’, ‘No’ and ‘Maybe’) that can be selected by an user. Using categorical vectorization of the answer keys and the method of hierarchical clustering^19^ (see “Methods”), we identify three clusters of people, indicating the existence of three major subpopulations in the society: Unaware (*U*) class who are yet to receive the message or the campaign, Broadcaster (*B*) class who have the potential to spread the campaign and Inert (*I*) class who are willingly or unwillingly not taking part in the campaign, though they have come across it at least once. The result is shown in form of a dendogram in Fig. 1a, considering the thresholding by black dashed line. We also note that 56% of the people who received a marketing message decided to forward it to their friends for some rewards/benefits, and eventually 93% of them stopped their participation in the campaign, which depicts the gradual picture of an Unaware becoming a Broadcaster, before finally turning into an Inert. Thus, the skeleton of the classical epidemiological model assumed by the previous researchers^11,12^ also emerges out.

Analysing further we see, in spite of having three major stand-points about VM campaigns, people can be further segregated into smaller sub-clusters based on different mentalities and activity level. We detect five such sub-clusters (threshold below the magenta line, Fig. 1a), which could be named according to their activities, as shown in Table 1. From the dendogram, we see that Inert class is a complex subpopulation as it is a combination of Rigid Inerts (who are strongly against bulk, unsolicited mailing, and thus, referral marketing, in general), Casual Inerts (who became Inert due to lack of relevance, safety, ease of share or profit) and Forgetful Inerts (who have all intentions similar to Broadcasters, except for being forgetful, they missed their participation in VM diffusion). The importance of these sub-clusters becomes evident as we look for more complex interactions that are very much probable on a social network, beyond the simple linear flow identified so far.

**Table 1:** Sub-clusters and subpopulations identified through hierarchical clustering. Five sub-clusters and their assigned titles based on polar responses are described in the first two columns. The central cluster of the dendogram in Fig. 1a with thresholding at magenta line corresponds to the Unaware class. The rest of the four clusters, red, green, violet and yellow correspond to broadcaster, forgetful inert, casual inert and rigid inert respectively. The major subpopulations (for the dendogram in Fig. 1a with thresholding at black line) are shown in the last column.

Population diversity and detection of major sub-populations		
Sub-clusters detected on the basis of comments	People category	Subpopulation
People who are not aware about a viral offer	Unaware	Unaware (U)
People who happily forward marketing messages for availing promotional product offer	Broadcaster (B)	Broadcaster (B)
People who received and were interested about the offer, but did not uses it, mostly due to forgetfulness.	Forgetful Inert ($I_F$)	Inert (I)
People who received, but are not sharing the marketing messages due to lack of relevance, safety, ease of share or profit	Casually Inert ($I_C$)	
People who are fearful about associated risks, and intentionally avoid bulk, unsolicited mailing.	Rigidly inert ($I_R$)	

### Identification of interactions from open ended questions

From the investigation of polar questions, we get to verify the basic structure of the epidemiological model. Being a referral marketing campaign, it is also evident that Unaware can become a Broadcaster, only if another Broadcaster influences him/her, making this an interactive or induced transition, which increases nonlinearity of the dynamics. To acquire further knowledge about the mutual interactions in the population and validate the model structure derived so far, we now take the open ended and semi-open ended questions into account using different language processing techniques which are explained in “Methods”. Here we refer to questions where users were asked to explain/ justify certain answers, or, where users could add answers other than the choices provided with the respective questions. A series of data pre-processings (mentioned in “Methods”) are applied on the collected data to capture the user-sentiments and the probable factors that were not considered by the surveyors while circulating the survey. Assuming the responses as mixture of different sentiments and factors that control the engagement of an individual in a VM campaign, we performed the LDA analysis on the preprocessed data. The LDA clearly shows that for decision making, the participants considered different factors like security (T2 and T4 in Fig. 1b), amount of profit (T7 in Fig. 1b), ease of sharing (T8 in Fig. 1b) and personal trust on the recommender as well as on the brand (T9 in Fig. 1) to forward or reject a received viral offer. Once the major topics of customer’s concerns are detected, we performed a systematic analysis of underlying causalities of the responses to conclusively estimate the correlation between these responses and the user activities. When a respondent took a step, or arrived at a conclusion about a marketing campaign, be it positive or negative, the action can be either spontaneous, or induced, depending on the influence of others. In Linguistics^20^, capturing this influence from the structure of the sentence is well-constructed through the concept of ‘Causation’. Following Shopen^20^, we redefine some basic phrases related to causation in the context of the problem we are working on:

- Agent: Someone, any member/person of the relevant social circle, who influences (i.e., actively or passively induces) the respondent to carry out an act.
- Agentive transition: A change of respondent’s perspective occurring through active influence of an external Agent.
- Inducive transition: A change of respondent’s perspective occurring through passive and more subtle influence of an external Agent.
- Autonomous transition: A change of respondent’s perspective occurring without any external agentive influence, the agent being the respondent himself/herself.

**Figure 2:**
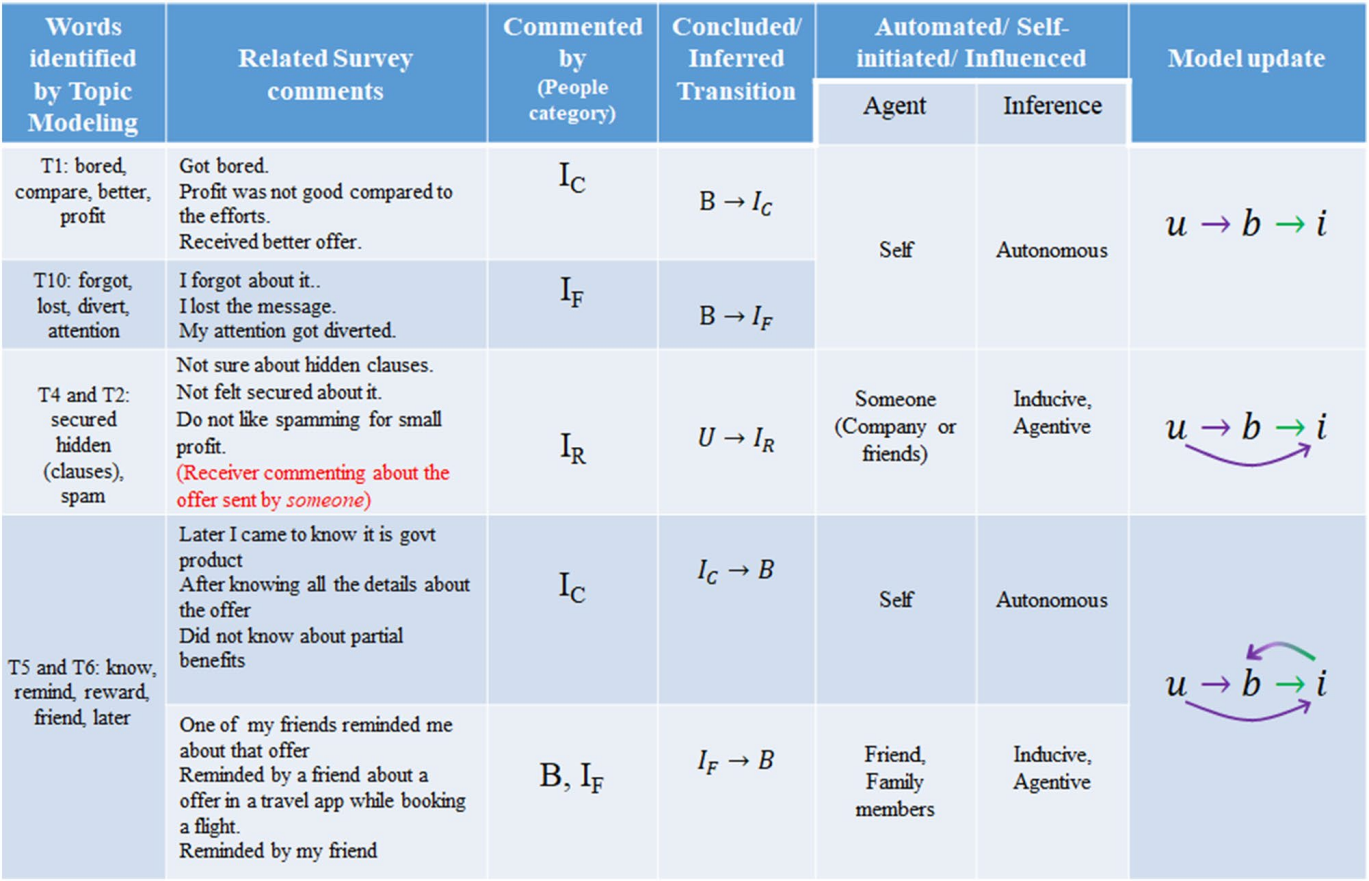
Causation analyses of survey data: this sums up the results of Causation analysis for important survey topics. Words, corresponding sentences from major topics, people category (based on clustering analysis) and inferred transitions are mentioned in the first four columns. Selected sentences are analysed on the grounds of Inducive/Agentive/Autonomous transitions as mentioned in “Identification of interactions from open ended questions”. In each step the corresponding model update has been shown in the last column. The violet (green) arrows denote Inducive/ Agentive (Autonomous) transitions which give rise to nonlinear (linear) terms in Eq. 1. The green-violet arrow indicates co-occurrence of Autonomous as well as Inducive transitions.

With this framework, we randomly selected up to 5 sentences for each of the words having top five scores in a topic, and analysed the sentences for all the topics which are found from the open-ended questions after LDA analysis. For these sentences, we performed a Causation analysis and exhibited a brief summary of the results in Fig. 2. Identifying the precise state change and drawing inference about its causation (Autonomous, Inducive or Agentive, as pointed out in column 6 of Fig. 2), we conclude about the contribution of the transitions in terms of linear or nonlinear factors in the dynamics.

### Important factors driving customer motivation

Through our rigorous analysis of the survey data, we arrive at some major conclusions regarding the driving factors of VM campaigns, which reflect customer’s perspective about marketing messages. We note that these observations arising from the outputs of the hierarchical clustering, LDA and causation analysis are also aligned with the findings of several recent surveys^14–16,21,22^.

#### Inherent aversion: rigidly inert

We note that the aversion towards bulk messages, possibly with fake commitments, has constructed the major theme of T2 and T4 in Fig. 1b. Our clustering study in dendogram Fig. 1a and Table 1 also point out that rigid inert, $I_R$ sub-cluster shows substantially different behavior from casual inerts, $I_C$. It is important to point out that the repulsion of users towards spam mails came out as an emergent theme from the spontaneous responses, while the interviewer never mentioned this idea in the questions. Support to our study can also be provided from several survey-based studies where the participants clearly mentioned that they often confuse the marketing mails with the spams, and associate a feeling of fear, intrusion, irritation, invasion of privacy and general security concerns with unsolicited marketing e-mails and mobile messages^15,17,22–24^. Thus, based on our observation, we conclude that, for a realistic modeling, behaviour of a sub-cluster among participants must be considered, who have a rigid repulsion against contextually blind automated bulk mailing, and thus, viral marketing messages, in general. A direct conversion from Unaware to Inert, immediately after encountering the marketing message, takes into account of this sub-cluster.

#### Brand trust

In marketing campaign propagation, the brand name plays a crucial role^25–28^. People comfortably share marketing messages from popular brands which can attract substantial numbers of prospective consumers. Research studies^29,30^ have correctly pointed out that in this era of seamless social persona, people understand the necessity to stand behind their words, and thus they inherently tend to believe reputed brands from the aspects of privacy and authentic engagement. Analysing the survey, we also detect that the topic T9 (Fig. 1b) associates the word ‘brand’, with words like ‘family’, ‘friend’ and ‘trust’. This shows that people need to ‘trust’ the person who is referring the product (e.g., ‘family’ or ‘friend’) as well as the brand-names while participating in referral marketing. We consider this as an important observation which should be incorporated in the model of campaign diffusion, by introducing a *probability factor* proportional to the brand reputation and popularity in the mass. This will directly contribute in making people interested about the campaign in the first place, if the brand is trustworthy, and will also nudge them to get back to the product, if they become inert eventually.

#### Remembering and reminding

Another important aspect that we notice in the survey^14^ is the distinctive reasons that can make a person inert about an advertisement campaign. People who lost interest, got bored or doubtful (due to low profit-to-effort ratio), denoted by sub-cluster $I_C$ in Table 1 can be intrigued by an authentic success story of the product/campaign. Words associated with T6 in Fig. 1 show that the strongest force to bring them back to broadcasting activity, is knowledge and relevant information. On the other hand, people who forgot or got diverted for some reason, denoted by sub-cluster $I_F$ in Table 1 could be brought back to the active group by a little reminder, as they will not need much persuasion. So, we conclude that while modeling the diffusion process mathematically, we must consider the complexity of these relapses. Strategically designed retargeting emails from the company^31^, informative advertisements and catchy slogans that motivate immediate consumer behavior^14,21,32^ could be one of the ways for the *I* class to return to the *B* state. Here the transition is not influenced by another individual, making this an Autonomous transition (see Fig. 2). On the other hand, reminders from friends^22^ or participation in recent discussions about a particular product in their own social circle^33^ (commonly termed as *buzz*) can tempt the inerts to become active again. This happens due to direct influence of peers making it an Inducive/Agentive transition (see Fig. 2). The brand name will also play a crucial role here as people avoid sending provocative marketing messages from lesser known advertisers to friends repetitively^16,28^.

## Data-driven model: formulation and results

**Figure 3:**
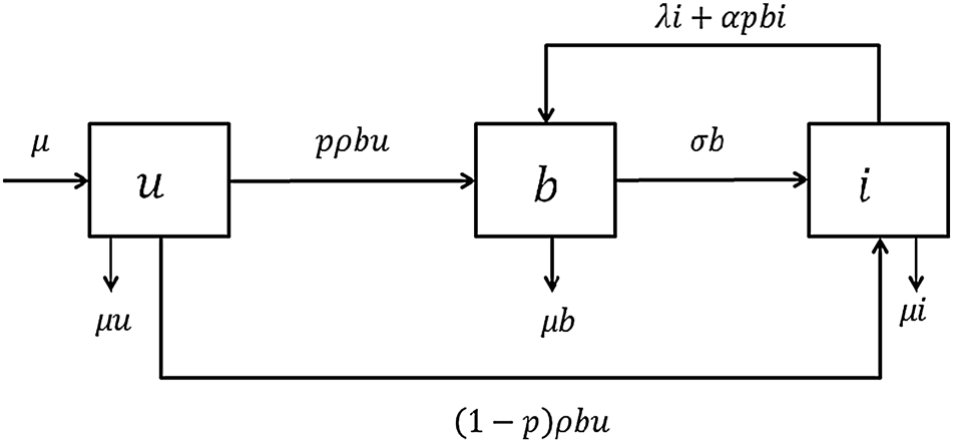
A schematic diagram of the proposed model. The arrows along with the parameters indicate the possible transitions from one state to other and the rate of transitions respectively.

### Formulation of model based on data analyses

Considering the identified subpopulations, transitions, nonlinearities and parameters in “Quantitative and qualitative analyses of survey data”, we model the dynamics of the system as shown in Fig. 3. As suggested by our clustering results, in our mean field analysis, we consider three non-overlapping subpopulations *U*, *B*, and *I* such that the total population $T=U+B+I$. We assume that at a rate $\rho$, a broadcaster spreads the message to a member from unaware class, which creates new prospective broadcasters. We consider that whenever a broadcaster sends the referral message to an unaware individual, unaware moves to broadcaster class with probability *p* and to inert class with probability $(1-p)$. We denote *p* as the trust parameter, which assumes a high value if the campaign is from a trusted brand or the message comes from a trusted member. The impact, or acceptability of the campaign to the unaware community is accounted using this probability parameter, *p*. Based on our discussions in “Inherent aversion: rigidly inert”, we take into account of the fact that some of the people from unaware class might have an inherent aversion to *spam-like* messages, and decide to ignore it straightaway. Messages from not so trustworthy brand or members increases the value of $(1-p)$.

Eventually, at a rate $\sigma$, the broadcasters either forget or lose interest about the campaign, and become inert. As discussed in Fig. 2, the possibility of relapse to the broadcaster state is complex. To justify all these possible transitions from *I* to *B*, we have included two feedbacks from inert class to broadcaster: one, a linear transition from inert to broadcaster with rate $\lambda i$, and another, a nonlinear interaction-driven transition. Considering the observations discussed in “Important factors driving customer motivation”, we decide that nonlinear relapse should depend on how well-known the brand is, and thus, we incorporate the probability *p*, the brand-trust parameter, in this relapse rate, considering the term as $\alpha ' b i$, while $\alpha '=\alpha p$, where $\alpha$ is the original relapse rate, and *p* again takes care of the acceptability of the advertisement to a person.

In practical scenarios, people enter and leave the population. To include this factor, we have introduced birth and death in our model. Both birth and death rates are kept equal to $\mu$, so that a fixed population size can be maintained^12^. For a particular VM dynamics, birth and death can be viewed as events when people join or leave a particular social platform where the campaign is going on. Considering numbers of unaware, broadcaster and inert individuals as continuously varying quantities, switching between subpopulations can be modelled by the following set of coupled ordinary differential equations:

1 $$\begin{aligned} u'= \,&{} \mu - \rho b u-\mu u \nonumber \\ b'= \,&{} p \rho b u+\lambda i+\alpha ' b i-\sigma b -\mu b \nonumber \\ i'=\, &{} \sigma b+(1-p) \rho b u -\lambda i -\alpha ' b i-\mu i \; \end{aligned}$$

where, *u*, *b*, *i* are normalized fraction belonging to each class such that $u+b+i=1$, $\alpha '=\alpha p$, and the rest of the parameters have significance as depicted in Fig. 3.

### Mean field study

**Figure 4:**
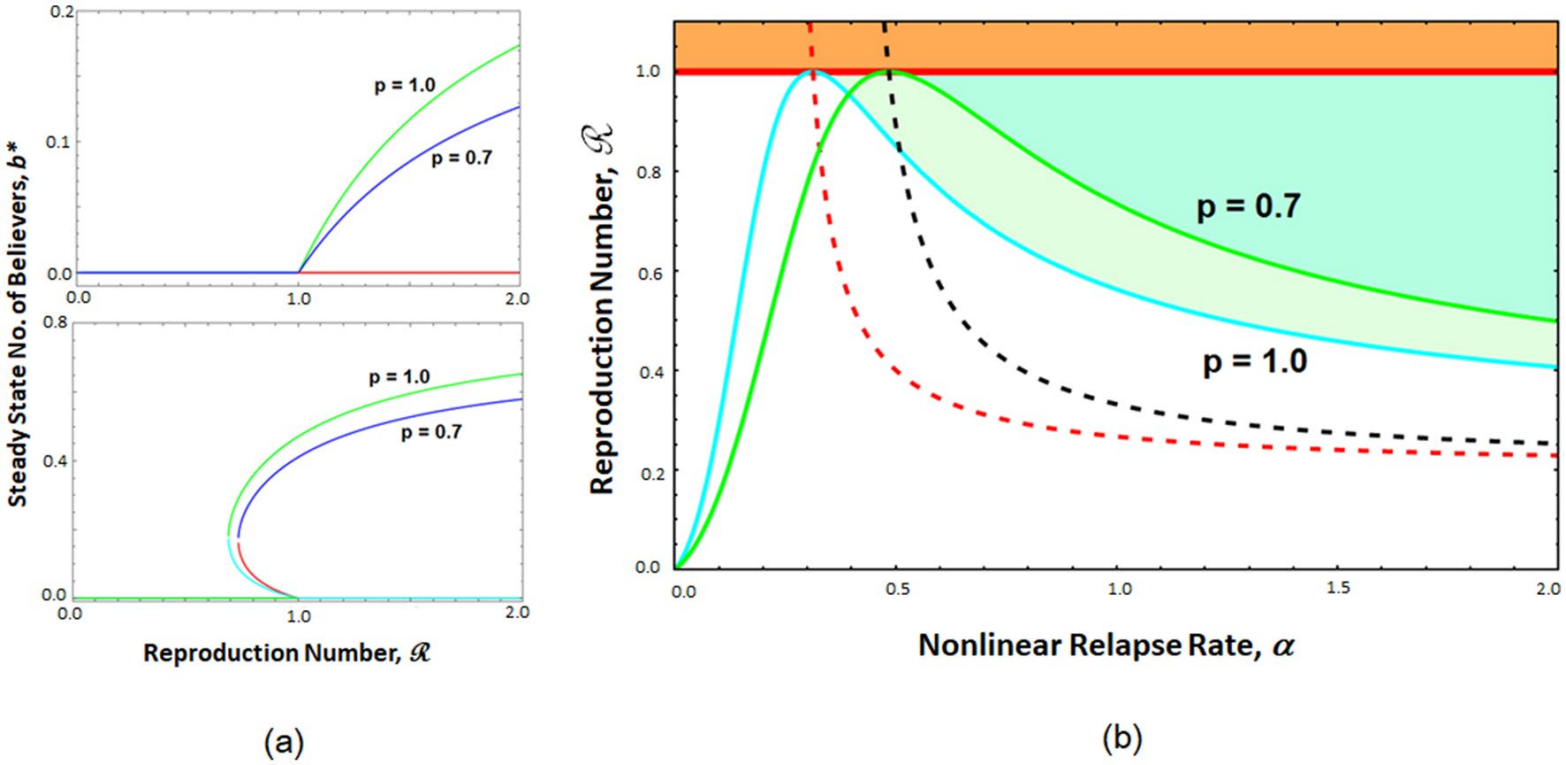
(**a**) Variation in steady state fraction of *b* with reproduction number ${{\mathscr{R}}}$ for (upper panel) $\alpha =0.1$, when only a single epidemic state persists beyond ${{\mathscr{R}}}=1$ and for (lower panel) $\alpha =1$, when bistability can be observed in range ${{\mathscr{R}}}_c$ to 1. Parameter values are $\sigma =0.2$, $\lambda =0.02$, and $\mu =0.05$. In these figures, green (and blue) lines indicate stable solutions while cyan (and red) lines indicate unstable solutions for $p=1$ (and $p=0.7$ respectively). For these parameter values, we calculated ${{\mathscr{R}}}_c=0.562$ (and ${{\mathscr{R}}}_c=0.594$) for $p=1$ (and $p=0.7$ respectively) using Eq. 6. (**b**) Phase diagram of the model in $\alpha -{{\mathscr{R}}}$ space for $\sigma =0.2$, $\lambda =0.02$ and $\mu =0.05$. The red line indicates ${{\mathscr{R}}}=1$. The region filled with orange color beyond ${{\mathscr{R}}}=1$ always exhibits monostable endemic state. The cyan (and green) line indicates ${{\mathscr{R}}}_c$, while the red (and black) dashed line indicates $\alpha _{th}$ for $p=1.0$ (and $p=0.7$). The white region, where $\alpha <\alpha _{th}$, exhibits monostable VM free state. In the green shaded region for $p=1.0$ (and darker green shaded region for $p=0.7$), $\alpha >\alpha _{th}$, ${{\mathscr{R}}}>{{\mathscr{R}}}_c$ and ${{\mathscr{R}}}<1$. Thus, this area exhibits bistability, where either VM free state or the endemic state is chosen by the system depending upon the initial state.

#### Reproduction number

Reproduction number for a marketing message propagation can be defined as the expected number of secondary broadcasters produced by typically a single person, who is recommending a product to his peers in a completely susceptible population. For simplistic models of mathematical epidemiology, this quantity defines the epidemic threshold of a particular infection. As found in the “Mean field study: equilibrium analysis”, reproduction number of the model is

2 $$\begin{aligned}{{\mathscr{R}}}= \frac{\rho (\lambda +\mu p)}{\mu (\lambda +\mu +\sigma )} \end{aligned}$$

From Eq. 2, we can observe that, for smaller value of *p*, a larger value of $\rho$ is required to satisfy the basic condition for an epidemic to spread, i.e., ${{\mathscr{R}}}>1$. In a practical sense, it means that if more people are switching directly to inert class from unaware class, may be for a lesser known brand, more marketing effort will be required to attain an endemic steady state. The sensitivities of ${{\mathscr{R}}}$ for various parameters are as follows:

3 $$\begin{aligned}\Gamma ^{{{\mathscr{R}}}}_{\rho }&=1 ;\;\;\; \Gamma ^{{{\mathscr{R}}}}_{p}=\mu p;\\ \Gamma ^{{{\mathscr{R}}}}_{\sigma }&=-\frac{\sigma }{(\lambda +\mu +\sigma )};\\ \;\;\; \Gamma ^{{{\mathscr{R}}}}_{\lambda }&=\frac{\lambda }{(\lambda +\mu p)} \frac{\sigma +\mu (1-p)}{(\lambda +\mu +\sigma )}; \nonumber \\\Gamma ^{{{\mathscr{R}}}}_{\mu }&=-\left( \frac{\lambda }{\lambda +\mu p}+\frac{\mu }{\lambda +\mu +\sigma }\right) \cdot \end{aligned}$$

It can be observed that ${{\mathscr{R}}}$ changes linearly with $\rho$. Thus, in our experiments, we change $\rho$ to vary ${{\mathscr{R}}}$ keeping other parameters fixed to analyze system dynamics and bifurcation. Positive value of $\Gamma ^{{{\mathscr{R}}}}_{p}$ also gives us the indication that brand reputation plays an important role in the final steady state values.

#### Bifurcation and its significance

The relapse rate happens to be a very important factor that defines the nature of the dynamics, as the model exhibits different behavior for smaller and larger value of $\alpha$ (discussed in “Mean field study: equilibrium analysis”). In Fig. 4a, we plot the steady-state fraction of class *B* for two different values of $\alpha$. To highlight the impact of the parameter *p*, results have been shown for $p=1$ and $p=0.7$. The upper panel of Fig. 4a shows the results for $\alpha =0.1$, a smaller relapse rate. This depicts that though the epidemic occurs after ${{\mathscr{R}}}_c=1$, the endemic steady-state fraction of broadcasters, $b^\star$, reduces as the value of *p* decreases. For a smaller value of *p*, the probability of an unaware individual to move to broadcaster class will be less, and more people will be moving from unaware class to inert class directly. As the relapse rate, $\alpha$ is also small and it is scaled with *p* as well, switching from inert to broadcaster will take place at a smaller rate. Reduction in broadcaster fraction, $b^\star$ can be attributed to the combined effect of these two parameters. It shows the effect due to an less appealing or less trusted advertisement campaign (smaller *p*), where no substantial efforts have been invested in ensuring relapse of lost customers (smaller $\alpha$).

In the lower panel of Fig. 4a, we can see that for a higher value of $\alpha$, a drastically different behavior can be observed. In this case, the significant change can be detected in the region ${{\mathscr{R}}}<1$, where we can see the emergence of a region of bistability. It clearly demonstrates that an endemic state, where prevalence of the viral campaign in ensured in the population, can even occur for ${{\mathscr{R}}}<1$ within a critical value of ${{\mathscr{R}}}={{\mathscr{R}}}_c$. Even when *p* decreases bistability still persists, suggesting that sustainability of the campaign can even be achieved for less appealing advertisements, *iff*, a significantly high relapse or regaining of inert customers can be accomplished. For any $p<1$, although a fraction of unaware population is coming directly to the inert class, the large value of $\alpha$ brings them back to the broadcaster class establishing endemic state till ${{\mathscr{R}}}={{\mathscr{R}}}_c$, which is markedly less than 1. We will proceed to figure out the expression of ${{\mathscr{R}}}_c$ in the next section.

#### Conditions for bistability

Bistability observed in our model system implies dependence of system dynamics on history (i.e., initial state of the population), which is commonly termed as hysteresis^34^. We have shown in “Mean field study: equilibrium analysis” that bistable region (multiple stable steady state solutions) may exist when ${{\mathscr{R}}}<1$, for larger values of $\alpha$. There are two conditions that must be satisfied to ensure bistability through the coefficients in Eq. 14.

- **Limiting value of induced relapse**, $\alpha$: Equating the coefficient *m* of Eq. 14 to zero, we found threshold value of $\alpha$
  4 $$\begin{aligned} \alpha _{th}= \frac{\rho (\sigma +\mu +\lambda )}{p(\rho -\mu )} \cdot \end{aligned}$$
- **Limiting value of infectious contact**, $\rho$: Again from Eq. 14, the critical value of $\rho$ can be calculated by equating $m^{2}-4ln$ to zero and is given by
  5 $$\begin{aligned} \rho _{c}= \frac{\alpha ' \mu }{(\alpha '+\lambda +\mu +\sigma )-2\sqrt{\alpha '(\mu -p\mu +\sigma )}}\cdot \; \end{aligned}$$
  Substituting $\rho _{c}$ in place of $\rho$ in the expression of reproduction number ${{\mathscr{R}}}$ (Eq. 2) gives the critical value of reproduction number ${{\mathscr{R}}}_{c}$ as
  6 $$\begin{aligned}{{\mathscr{R}}}_{c}= {} \frac{\rho _{c}(\lambda +\mu p)}{\mu (\lambda +\mu +\sigma )} = \frac{\alpha ' \mu }{(\alpha '+\lambda +\mu +\sigma )-2\sqrt{\alpha '(\mu -p\mu +\sigma )}} \frac{(\lambda +\mu p)}{\mu (\lambda +\mu +\sigma )}\cdot \end{aligned}$$

Figure 4b depicts the dynamical behaviour in form of a phase diagram in $\alpha -{{\mathscr{R}}}$ space for $\sigma =0.2$, $\lambda =0.02$ and $\mu =0.05$. We point out that the region of bistability here indicates the sustainability of the advertisement campaign in a population even when ${{\mathscr{R}}}<1$, i.e., the infective contacts are not that effective. We note that the region of bistability shrinks as *p*, the brand-trust parameter decreases. It clearly points out that well-known brands are much more prone to have resonant stories in social media due to their established brand-value.

### Graph-theoretical analysis

#### Model dynamics on networks

In contrast to mean-field approach, diffusion in networks will be dependent on the degree distribution of the network. We denote with $u_{k}$, $b_{k}$, and $i_{k}$ the fraction of unaware, broadcaster and inert nodes with degree *k*. Nodes having same degree are considered to behave in same fashion. Using this degree block approximation^2^, differential equations for evolution of degree based compartments of different class will be

7 $$\begin{aligned} u_k'= \, &{} \mu - \rho _{n} k u_{k} \Theta _{b}-\mu u_{k} \nonumber \\ b_k'=\, &{} p\rho _{n} k u_{k} \Theta _{b} +\lambda i_{k}+\alpha _{n} k p i_{k} \Theta _{b}-(\sigma +\mu ) b_{k}\; \nonumber \\ i_k'= \, &{} \sigma b_{k} +(1-p) \rho _{n} k u_{k} \Theta _{b}- \lambda i_{k}-\alpha _{n} k p i_{k} \Theta _{b} - \mu i_{k}\; \end{aligned}$$

We are considering $\rho _{n}$ to be the rate at which a broadcaster spreads the information to an unaware neighbor. Similarly, $\alpha _{n}$ is the relapse rate influenced by the neighbors. Subscript ‘*n*’ in both these symbols signify network setting and they are counterpart of $\rho$ and $\alpha$ used in homogeneous setting. $\Theta _{b}$ is the density function which gives probability of broadcasters around a node. In a general scenario, density function of any class around a node depends on degree of the node but for uncorrelated network, it is independent of degree *k* and is given by $\Theta _{b} =\sum _{k} \frac{k p_{k} b_{k}}{\left\langle k \right\rangle }$.

Multiplying all three equations of Eq. 7 by $\frac{k p_{k}}{\left\langle k \right\rangle }$ and then performing summation over *k*, we get

8 $$\begin{aligned} \Theta _{u}'&=\, {} \sum _{k} \frac{k p_{k}}{\left\langle k \right\rangle }\mu - \rho _{n} \sum _{k} \frac{k^{2} p_{k}}{\left\langle k \right\rangle } u_{k} \Theta _{b}-\mu \sum _{k} \frac{k p_{k}}{\left\langle k \right\rangle } u_{k} \nonumber \\ \Theta _{b}'&= \, {} p \rho _{n} \sum _{k} \frac{k^{2} p_{k}}{\left\langle k \right\rangle } u_{k} \Theta _{b}+\lambda \sum _{k} \frac{k p_{k}}{\left\langle k \right\rangle } i_{k} +\alpha _{n} p \sum _{k} \frac{k^{2} p_{k}}{\left\langle k \right\rangle } i_{k} \Theta _{b}-(\sigma +\mu ) \sum _{k} \frac{k p_{k}}{\left\langle k \right\rangle } b_{k} \nonumber \\ \Theta _{i}'&=\, {} \sigma \sum _{k} \frac{k p_{k}}{\left\langle k \right\rangle } b_{k}+(1-p) \rho _{n} \sum _{k} \frac{k^{2} p_{k}}{\left\langle k \right\rangle } u_{k} \Theta _{b}-\lambda \sum _{k} \frac{k p_{k}}{\left\langle k \right\rangle } i_{k}\\&\,\,\,\,\,\,\,\,\,\,\,\,\,\,\,\,\,\,\,\,\,\, -\alpha _{n} p \sum _{k} \frac{k^{2} p_{k}}{\left\langle k \right\rangle } i_{k} \Theta _{b}-\mu \sum _{k} \frac{k p_{k}}{\left\langle k \right\rangle } i_{k} \end{aligned}$$

We solve Eq. 8 at initial phase of the campaign and at the steady state to get the epidemiological threshold for the heterogeneous structure.

#### Propagation at initial state

Solving simultaneous linear differential equations Eq. 17 and Eq.  18, we have

9 $$\begin{aligned} \frac{\mathrm{d^{2}} \Theta _{b}}{\mathrm{d} t^{2}}-(C_{1}+C_{4})\frac{\mathrm{d} \Theta _{b}}{\mathrm{d} t}+(C_{1} C_{4} -C_{2} C_{3})\Theta _{b}=0 \; \end{aligned}$$

As clear from the form of the equation, $\Theta _{b}$ will be the summation of two exponential, exponent of which depends on roots of auxiliary equation of the differential Eq. 9. For campaign to spread, $\Theta _{b}$ needs to be an increasing function in time. Thus, we have the condition

10 $$\begin{aligned} \frac{\rho _{n}}{\mu } \frac{\lambda + p \mu }{ (\sigma +\lambda +\mu )}>\frac{\langle k \rangle }{\langle k^{2} \rangle } \end{aligned}$$

Replacing $\rho _{n}$ by $\frac{\rho }{\langle k \rangle }$ the condition modifies to

11 $$\begin{aligned} \frac{\rho }{\mu } \frac{(\lambda + p \mu )}{ (\sigma +\lambda +\mu )}={{\mathscr{R}}}>\frac{\langle k \rangle ^2}{\langle k^{2} \rangle } \cdot \end{aligned}$$

Left-hand side of the inequality is the reproduction number ${{\mathscr{R}}}$ of the homogeneous model and right-hand side is $\frac{\langle k \rangle ^2}{\langle k^{2} \rangle }$ which depends on average degree and average of square of individual degrees of the nodes in the network. The exact value of the expression will depend on the type of network. The expectation of $k^2$ for a random network with Poisson degree distribution is $\left\langle k^{2} \right\rangle =\left\langle k \right\rangle \left( \left\langle k \right\rangle +1 \right)$.

Using the expression for $$\left\langle k^{2} \right\rangle$$ in Eq. 11, we get $${{\mathscr{R}}}>\frac{\left\langle k \right\rangle }{\langle k \rangle +1}$$ which can be further approximated by $${{\mathscr{R}}}>1$$ if $$\left\langle k \right\rangle>>1$$. It is important to observe that from the mean field analysis, we achieved the same condition of epidemiological outbreak. If we consider a scale-free network, its degree distribution can be written as $$p(k)=Bk^{-\gamma }$$. Using this degree distribution, we estimate the average degree as $$\left\langle k \right\rangle =B\frac{1}{\gamma }l^{2-\gamma }$$, and average of degree square as $$\left\langle k^{2}\right\rangle \approx B\int _{l}^{\infty }k^{2-\gamma }dk$$. When $$\gamma \in (2,3]$$; $$(2-\gamma )$$ is in range of $$[-1,0)$$; $$\langle k^{2} \rangle$$ diverges, and for $$\gamma >3$$, $$\left\langle k^{2} \right\rangle$$ is finite. In other words, in the range $$\gamma \in (2,3]$$, there is no threshold for epidemiological outbreak. For other values of $$\gamma$$, we will observe similar behaviour like random network with different diffusion rate.

**Figure 5:**
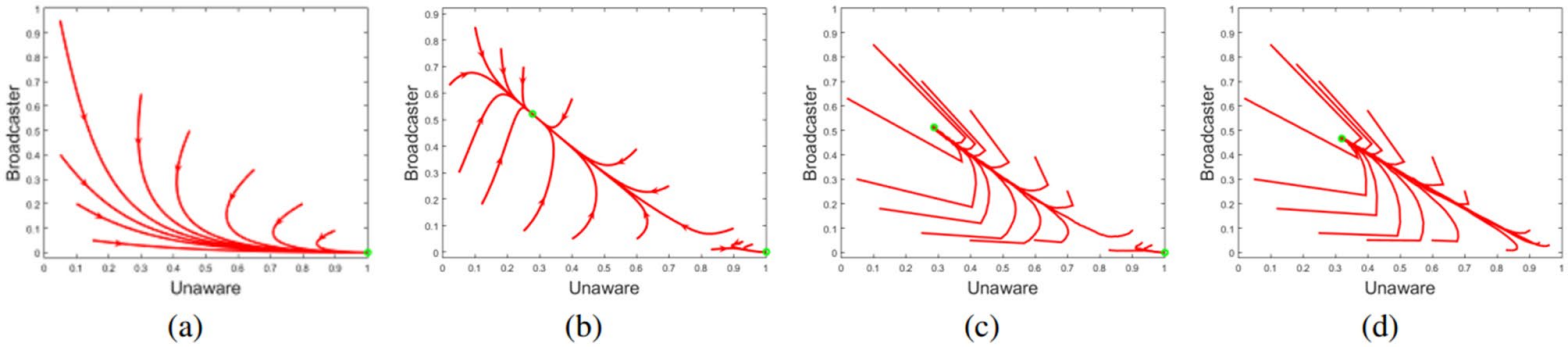
Numerical simulation of convergence to the steady state for different initial conditions with parameter values $\mu$= 0.05, $\rho = 0.25$, $\sigma = 0.2$, $\lambda = 0.02$, $p=0.7$, and (**a**) $\alpha =0.1$ for a homogeneous system with a single campaign free steady state; (**b**) $\alpha =1$ for a homogeneous system with bistable steady states; temporal variation of *u* and *b* with different initial conditions for equivalent parameter regime as for (**b**) in (**c**) random network and (**d**) scale-free network. In all of these figures, X and Y coordinates of the initial point of any flow represents the initial fractional population of unaware and broadcaster class of the population.

#### Steady state network analysis

As discussed in “Steady state condition in network propagation”, in steady state, the expression of $\Theta _{b}$ becomes a consistency equation, i.e., we have $\Theta _{b}= f(\Theta _{b})$. At $\Theta _{b}=0$, $f(\Theta _{b})$ is also zero. Hence $\Theta _{b}= 0$ is a solution of the equation. Value of the function at $\Theta _{b}=1$ is

12 $$\begin{aligned} f(1)&=\frac{1}{\left\langle k \right\rangle } \sum _{k} \frac{p_{k} k^{2}\rho _{n}(\mu p+\lambda +\alpha _{n} k p)}{(\mu +\rho _{n}k)(\lambda +\mu +\sigma +\alpha _{n}k p)} \\ &= \frac{1}{\left\langle k \right\rangle } \sum _{k} \frac{p_{k} k}{(1+\frac{\mu }{\rho _{n} k})(1+\frac{\sigma +(1-p)\mu }{\lambda +\mu p+\alpha _{n}k p})} \end{aligned}$$

It is clear from the above expression that $f(1)<1$. To have another solution in the interval 0 to 1, slope of the function at $\Theta _{b}=0$ must be greater than 1.

$$\begin{aligned} \frac{\mathrm{d} f(\Theta _{b})}{\mathrm{d} \Theta _{b}} \Big |_{(\Theta _{b}=0)}= &{} \frac{1}{\left\langle k \right\rangle }\sum _{k} \frac{p_{k} k^{2} \rho _{n}(\mu p +\lambda ) }{\mu (\lambda +\mu +\sigma )} =\frac{\rho _{n}(\mu p +\lambda )}{\mu (\lambda +\mu +\sigma )} \frac{\left\langle k^{2} \right\rangle }{\left\langle k \right\rangle }\ge 1 \end{aligned}$$

After replacing $\rho _n$ by $\frac{\rho }{\left\langle k \right\rangle }$, we will get the same condition what we had in early stage analysis, i.e., $\frac{\rho }{\mu } \frac{(\lambda + p \mu )}{ (\sigma +\lambda +\mu )}={{\mathscr{R}}}>\frac{\langle k \rangle ^2}{\langle k^{2} \rangle }$.

### Numerical results

We have carried out the simulations for homogeneous as well as heterogeneous approach. Along with random and scale free networks, some real network structures have also been considered, including one constructed from our own referral experiment. To compare the results of deterministic mean-field model with network model, we select same set of parameters values in simulations.

#### Simulation of deterministic model

Depending upon the parameter values, system may lead to message-free state or endemic state. Two different cases for homogeneous setting have been shown in Fig 5a, b. As discussed for Eq. 4, bistability can be observed in the system for value of $\alpha$ greater than $\alpha _{th}$. It is observed in Fig. 5b, depending on initial fraction of different classes, system reaches to endemic or message-free equilibrium; this exhibits hysteresis.

**Table 2:** Important characteristics of different networks

Network characteristics	Hamster network	Email network	Jazz network	Recommendation network
Number of nodes	2,426	1,133	198	1,157
Number of edges	16,631	5,451	2,742	2,558
Average degree	13.71	9.624	27.7	4.422
Maximum degree	273	71	100	59
Power law exponent	2.46	6.77	5.27	1.34

#### Simulation over model networks

Parameter values for bistable case have been used to plot the results for both of the model networks, random as well as scale-free network in Fig. 5c, d respectively. Results of random network almost matches the findings of homogeneous model. Along with similar endemic steady-state values, bistability can also be observed in random network scenario of Fig. 5c. In case of scale-free network, endemic steady state values are not exactly same and maximum error in endemic steady-state fraction of a particular class is 5%. Under bistable parameter set, message-free steady state never appears and system leads to endemic steady state for every set of initial conditions. It can be observed in Fig. 5d where every flow terminates at endemic steady state. This observation is in alignment with our analytic result regarding absence of epidemic threshold in scale-free network as mentioned in “Propagation at initial state”.

**Figure 6:**
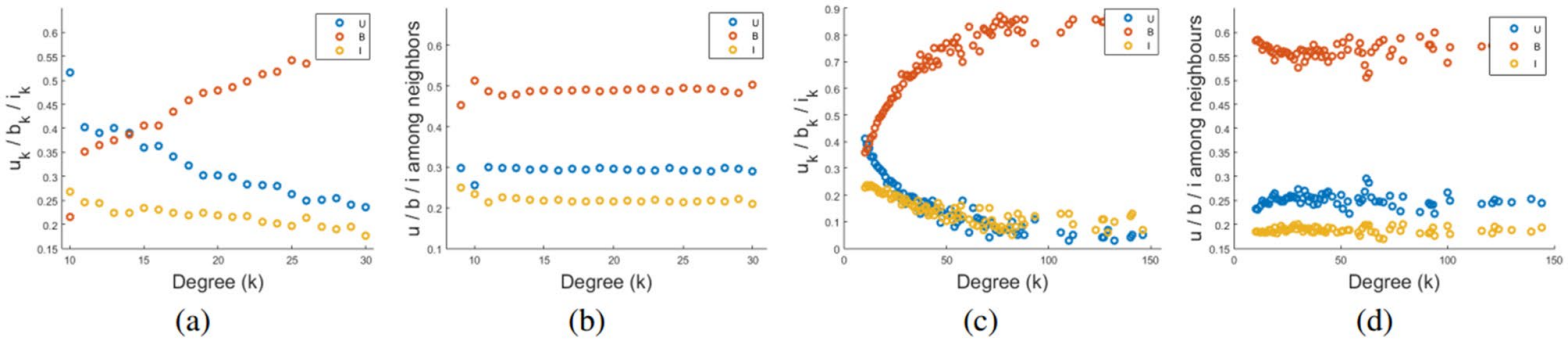
(**a**) $u_k$, $b_k$ and $i_k$ with respect to *k* at steady-state in random network; (**b**) fraction of *u*, *b* and *i* in the neighborhood of a node with degree *k* in random network; (**c**) $u_k$, $b_k$ and $i_k$ with respect to *k* at steady-state in scale-free network; (**d**) fraction of *u*, *b* and *i* in the neighborhood of a node with degree *k* in scale-free network.

Degree-wise steady state fraction $u_{k}$, $b_{k}$, and $i_{k}$ has been plotted for random and scale free networks in Fig. 6a, c respectively. Fraction of *u*, *b* and *i* in the neighborhood of a node of different degrees has also been plotted for both the networks in Fig. 6b, d respectively. A node with higher degree has higher probability to be in broadcaster class, in random as well as scale free network. For random network, the fraction of believers around a node is independent of its nodal degree, but for scale free network this fraction is not identical. It is again due to the heterogeneous structure of the network and presence of hubs in the network.

#### Simulation over real networks

The important parameters associated with some real networks (Hamster network, Email network and Jazz network) collected from KONECT database^35^ are tabulated in Table 2. We have used these networks for our simulation studies; the steady state fractions of unaware ($u^*$), broadcaster ($b^*$) and inert ($i^*$) for various networks are mentioned in Table 3; for comparison, results for homogeneous dynamics are also mentioned in that table.

**Table 3:** Comparison of steady state fractions in various networks, with the homogeneous dynamics $({{\mathscr{R}}}=0.64$)

Steady state fraction	Homogeneous setting	Random network	Scale-free network	Hamster network	Email network	Jazz network	Recommendation network
$u^*$	0.277	0.288	0.319	0.512	0.394	0.348	0.352
$b^*$	0.521	0.510	0.467	0.342	0.417	0.469	0.441
$i^*$	0.202	0.202	0.214	0.146	0.189	0.183	0.207

**Figure 7:**
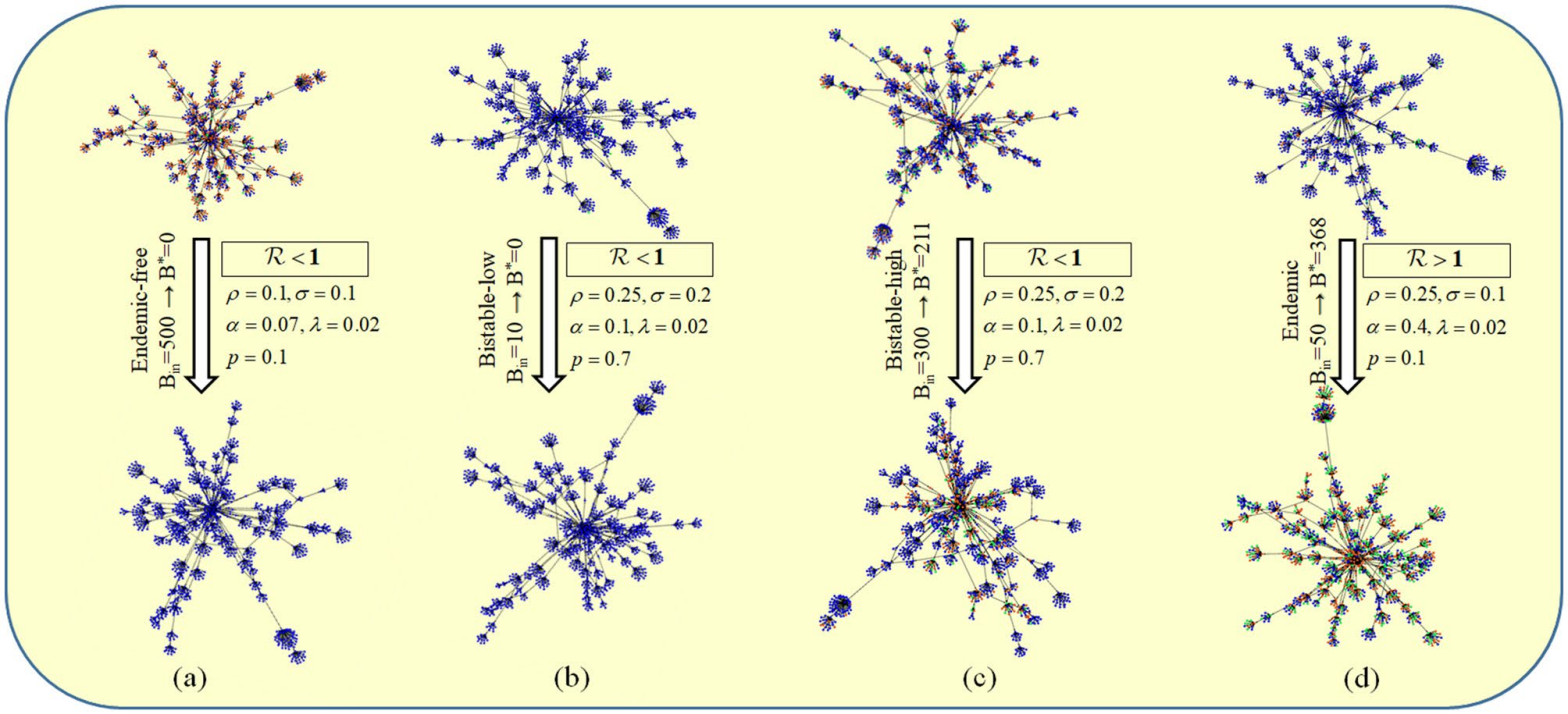
Different steady state transitions of the recommendation network. The top row indicates initial conditions of the network with different initializations and parameter values, and the bottom row indicates the final state of the network for the corresponding cases. Blue color indicates unaware nodes whereas orange and green indicate broadcaster and inert nodes. $B_{in}$ and $B^*$ indicate initial and final number of broadcasters. (**a**) When the parameters are selected from an endemic-free region, in the steady state, the population is always broadcaster free. In (**b**) and (**c**), parameters are selected from bistable region. Thus, when the network is initialized with small number of broadcaster it reaches to a broadcaster free state, but when initialized with more number of broadcasters, the final steady state is endemic even though ${{\mathscr{R}}}<1$. (**d**) The parameters are selected from an endemic region. So, even the initial number of broadcasters are small, the network always reaches to an endemic steady state.

Though the networks taken from KONECT database account for social connections, and each of them depicts contacts between people in a community, none of these networks are actually recommendation network. To better understand the referral flow, we perform an extensive experiment (see in “Methods”) to build up a recommendation network. After generating the recommendation network, we observe the final steady states of the network for different parameters and initialization as shown in Fig. 7, where we have shown three different cases. In Fig. 7a, we set the parameters such that ${{\mathscr{R}}}<{{\mathscr{R}}}_c<1$. In this case, even though we have 500 broadcasters initially (denoted by $B_{in}$ in the figure), in the final steady-state there is no broadcaster left. For Fig. 7b, c, we keep the parameters such that ${{\mathscr{R}}}_c<{{\mathscr{R}}}<1$, i.e., the parameter set is in the region of bistability. In this parameter setting, if we keep $B_{in}$ very low, the steady state becomes broadcaster free, however, if we start with sufficiently high number of broadcasters, we get endemic steady state even if ${{\mathscr{R}}}<1$. Here, Fig. 7b, c can be associated with the lower branch (cyan or red) and the upper branch (green or blue) of Fig. 4a. The lower branch and the upper branch is detected as mentioned in^36^. In the bistable region, different final states of the recommendation network for different initial conditions exhibits the presence of hysteresis. We also observe that, system’s propensity for the endemic state increases as ${{\mathscr{R}}}$ goes close to 1. For different real networks, we consider parameters equivalent to Fig. 4a (bottom) with ${{\mathscr{R}}}=0.64$ to observe the flow, and the final steady state fractions of $u^*$, $b^*$ and $i^*$ are compared in Table 3. It can be observed that even when ${{\mathscr{R}}}<1$, 41-51% of the population belong to the broadcaster class, indicating the survival of the campaign in steady state because of the bistable property.

## Summary and discussions

For a successful business, marketing is not only a supportive component, but it is one of the key ingredients^37,38^. In today’s digital environment, the goal of a marketing campaign might have grown beyond enticing a consumer to click on a product icon. Now the objective has expanded to reach maximum number of people and create ‘sustained engagement’ with the consumer^39^. Weber has correctly pointed out^40^ that to succeed in social media marketing, marketers must ‘talk *with*’ the customers, rather than ‘talk *at*’ them. Surely, a VM campaign provides an inexpensive, personal-level way to reach the customers, where the inherent ignorance or indifference of the customers can be handled well. But with more than 3 million active advertisers posting their ads on social networking sites like Facebook, to reach a bigger audience, it is essential that the campaign establishes substantially long iterations in the population. In last couple of years, marketers have gradually started to use retargeting to convert customers who had formerly window-shopped in their websites, or abandoned carts suddenly^31,41–44^. It has become a familiar part of our web-experience now-a-days to see hyperlinked photos of products that we have once browsed. But some recent works have demonstrated that severe privacy concern and cynicism among consumers is a straightforward effect of constant retargeting, which directly affects the buying intent^17,23,24,45^. Interestingly, two consecutive surveys in 2015 and 2017^46^ pointed out, that customers identify a brand as unprofessional, suspect superficiality, and finally email opt-out (or unsubscribe) in case of excessive frequency of promotional mails^47^.

In this paper, we show that, while one way for ensuring re-engagement could be repetitive posting of the campaign by the firm itself, a much more effective way could be by creating a framework where some people in the population always remain enthusiastic about the product, and act as ambassadors themselves. Our proposed model relies thoroughly on a survey data that deals with consumer mindset and outlook^14^, to prescribe ways to achieve sustainability for online campaigns. Using thorough analysis based on unsupervised hierarchical clustering, we also demonstrate that customers have an individuality which cannot be ignored; for example, inherent aversion towards marketing messages among a considerable percentage of people have to be considered. Our LDA topic modeling shows that, beside the creative aspects of the campaign, the brand loyalty and brand name also plays a very important role to increase the probability of positive reaction of a person towards a campaign message. Exhaustive investigation of our differential equation based quantitative model shows the nonlinear relapse rate, $\alpha$, plays a key role to safeguard the survival of the campaign. This parameter is an estimate of social-circle-driven retargeting; as a group of friends, families or colleagues often have similar interests as well as proximity, assuring the unresponsive inert customers about the authenticity, security and usefulness of the campaign becomes much more plausible through this way. We also show that bistability, which comes into picture due to the inherent nonlinearity of the dynamics, works as an opportunity for the firm to make the viral campaign maintain its endemic state, even in adversarial conditions. We have calculated and discussed the critical parameters that help to sustain the desired endemic state by exploiting the properties of bistability.

Exploring beyond the homogeneous system dynamics, we studied the system considering the network structures. Along with model networks, we simulated the dynamics on existing social networks as well as on our experimentally generated recommendation network, to show that regaining those who are not showing any interest despite being aware of the campaign, is extremely important; acquirement, retargeting, and win-back of these customers create the path towards the success of an online marketing campaign. As shown in Fig. 7c, in a favourable diffusion setting, the population may exhibit endemic state even though ${{\mathscr{R}}}<1$. As discussed in “Important factors driving customer motivation”, for newer brands (i.e., where the value of brand trust parameter *p* is not in favor), along with the lucrativeness of the offer, figuring out innovative ways to remind the unresponsive customers can be the key to success. With no brand history, these points must be included in their referral policy, otherwise an initial adverse acceptability of the brand might lead to the failure of the entire campaign process.

Beyond the findings we report in this paper, our methodology has potential to push forward the field of quantitative modelling of referral marketing dynamics. The brand-trust parameter *p* brings the idea of realistic decision making to quantitatively understand referral marketing psychology. This parameter is chosen to give an estimate of the perceived trust the community has for a particular brand, so that, without any hesitation, they can participate in a marketing campaign. With this idea of *perceived trust*, our referral marketing model can also be studied from the perspective of evolutionary game theory. In voluntary participation to a marketing campaign, an individual’s decision-making may depend on several factors such as the trust towards the brand, consequences after participation, incentives associated with a campaign and fear of phishing or spamming. In a future study, we will proceed to associate the dependence of decision making on the behavioural response of fellow community members, and model the problem from the point-of-view of the traditional social dilemma depicted by game theory^48^. This work is the first step which can connect the well-developed tools of vaccination games with the strategies of referral marketing policies causing a huge benefit for the marketing community.

Moreover, our work is the first of its kind, where we introduce unsupervised algorithms to understand customer psychology towards VM campaigns and implement it in form of a precise dynamical model which mimics epidemic-like behavior. Our proposed methodology is extremely promising in bridging the gap between social surveys and mathematical modelling, in general. Inside and outside the boundaries of marketing field, several social issues involving public psychology are commonly explored and researched by committed efforts of survey-based studies. On the other hand, mathematical models and computation based analysis have also shown great implications in understanding and predicting complex dynamical social systems. To our knowledge, there is no other study where a concrete methodology has been proposed with survey-based data giving direct inputs to model construction, not by heuristic arguments but by solid quantitative study through extensive language processing, which includes machine learning methodologies like hierarchical clustering and LDA. In our work, we have shown that even with simple yes/no questions, a systematic quantitative analysis can identify clusters in opinion. Combining open-ended survey responses, NLP based tools, mean-field study and network analysis, we put the pieces of a puzzle together which can offer a very general comprehensive methodology to mathematically and computationally analyse survey responses, make concrete predictions on behavioural reaction through mathematical models, and see the implications in real-life social networks.

## Methods

### Analyses of survey data using hierarchical clustering and LDA

NLP provides different ways to analyze textual data^49–52^. We briefly describe the process that we follow to extract the model structure and probable transitions from the user responses. The responses of the polar questions of the survey data of Ghosh et al.^14^ are taken as dataset where each individual has given answer to eight polar questions. Our task was to divide the population to sub-populations depending on the responses. To do so, first we convert the each polar response to vector, which gives us 8 vectors for each of the respondent. Then, we use Ward’s linkage^53^ to perform the hierarchical clustering on the dataset. As shown in Fig. 1a, the dendogram shows that there are three distinct sub-population present among the respondents, where each sub-population contains further sub-classes, i.e., group of people with different mentality but almost similar behaviours.

Next, we perform a series of preprocessing on the responses of the open-ended questions, which include removal of English stop words, punctuation and numbers^54^ from the data, lower-case conversion and lemmatization to have a standard representation of the text. To understand the correlation between these words, next, we perform topic modeling on the processed text data. Assuming the collection of responses to an open ended question as a corpus, where each response is a document, we individually apply Latent Dirichlet allocation (LDA)^55^ on each corpus, assuming that the responses cover one or more topics. Assuming each topic is defined as a distribution over words, the posterior probabilities given a document collection determines a decomposition of the collection into topics. If in a corpus ${\mathbf{D}}$ with *K* topics, we have *R* responses having up to *N* word tokens each from a vocabulary *V*, then each response has a *K* dimensional multinominal distribution $\theta _d$ over topics having a common Dirichlet prior $Dir(\mathbf{\alpha })$. Each of the topic has a *V* dimensional multinominal $\beta _k$ over words with a common symmetric Dirichlet prior $Dir(\eta )$. To estimate $\mathbf{\alpha }$, $\mathbf{\beta }$ from a corpus *D*, we maximize the log likelihood $\text{ln } P({\mathbf{D}}|\mathbf{\alpha },\mathbf{\beta })$. We have used ‘gensim’ and ‘nltk’ packages to implement LDA on our responses. The optimal number of topics in the LDA model is decided using the coherence score.

### Mean field study: equilibrium analysis

The system of Eq. 1 can have two kinds of equilibrium: a VM-free equilibrium $E_0$ (with the entire population being unaware), and an Endemic equilibrium $E^{\star }$ (with a finite percentage of broadcasters present in steady state). As there is no time evolution at equilibrium, all the components of $E^{\star }$ can be evaluated by equating $u'$, $b'$ and $i'$ of Eq.  1 to zero. While solving for $E^{\star }$, the first equation of system model, defined in Eq. 1, gives

13 $$\begin{aligned} u^{\star }=\frac{\mu }{\rho b^{\star }+\mu } \end{aligned}$$

Relevant substitutions from Eq. 13 and replacing $i^{\star }$ by $(1-b^{\star }-u^{\star })$, simple algebra results into $l(b^{\star })^{2}+mb^{\star }+n=0$, where

14 $$\begin{aligned} l= \,&{} \alpha p \rho \nonumber \\ m=\, &{} (\sigma \rho +\mu \rho +\lambda \rho +\alpha p \mu -\alpha p \rho ) \nonumber \; \\ n=\, &{} \mu (\sigma +\mu +\lambda )- \rho (\lambda +\mu p)\cdot \end{aligned}$$

Examining the coefficients, we conclude that *l* is always positive; *m* is positive for small values of $\alpha$, and *n* is positive or negative depending on whether $\frac{\rho (\lambda +\mu p)}{\mu (\lambda +\mu +\sigma )}={{\mathscr{R}}}$ is smaller or greater than 1. Two completely different steady state scenarios can arise:

**Case 1:** For negative *n* (i.e., ${{\mathscr{R}}}>1)$, the quadratic equation has a unique positive solution $b^{\star }_+$, as another solution $b^{\star }_-$ is always negative and so, unphysical, and there exists a unique endemic equilibrium $E^{\star }$ whenever ${{\mathscr{R}}}>1$.

**Case 2:** On the other hand, for positive *n* (i.e., ${{\mathscr{R}}}<1)$, the number of physical roots of the equation depends on the sign of *q*, and therefore, the nonlinear relapse parameter $\alpha$. Depending on this fact if $\alpha$ is high (or low), multiple (or no) endemic equilibria may exist.

For analysing stability of these equilibria, we consider Eq. 1 as

15 $$\begin{aligned} f_{1}=\, &{} \mu -\rho b u-\mu u \nonumber \\ f_{2}=\, &{} p \rho b u+\lambda i+\alpha p b i-\sigma b -\mu b \nonumber \\ f_{3}=\, &{} \sigma b+(1-p) \rho b u -\lambda i -\alpha p b i-\mu i \;\end{aligned}$$

and calculate the Jacobian of ${\mathbf{f}}$, where ${\mathbf{f}}=[f_1,f_2,f_3]$^13^.

### Parameter selection in network

Instead of same $\rho$ and $\alpha$ for every node, as considered in the mean-field analysis, the rates have been made proportional to degree of the nodes. $\rho _n$ and $\alpha _n$ are chosen to be $\frac{\rho }{ \langle k \rangle }$ and $\frac{\alpha }{ \langle k \rangle }$ respectively so that expected values of $\rho _n k$ and $\alpha _n k$ are $\rho$ and $\alpha$ used in mean-field analysis. It synchronizes the parameter set of both the approaches and allows us to compare the obtained results.

### Initial propagation in network

In initial phase of message spreading^56^, *b* and *i* can be approximated by zero and *u* by 1. Using these values in Eq. 8, we get

16 $$\begin{aligned} \Theta _{u}'= &{} \mu - \rho _{n} \frac{\left\langle k^{2} \right\rangle }{\left\langle k \right\rangle } \Theta _{b}-\mu \Theta _{u} \nonumber \\ \Theta _{b}'= &{} p \rho _{n} \frac{\left\langle k^{2} \right\rangle }{\left\langle k \right\rangle } \Theta _{b}+\lambda \Theta _{i}-(\sigma +\mu ) \Theta _{b} \; \nonumber \\ \Theta _{i}'= &{} \sigma \Theta _{b}+(1-p) \rho _{n} \frac{\left\langle k^{2} \right\rangle }{\left\langle k \right\rangle } \Theta _{b} -(\lambda +\mu ) \Theta _{i} \end{aligned}$$

Last two equations of the Eq.  16 forms a system of simultaneous linear differential equations with constant coefficients.

17 $$\begin{aligned} \frac{\mathrm{d}\Theta _{b} }{\mathrm{d} t}= &{} C_{1}\Theta _{b}+C_{2} \Theta _{i} \end{aligned}$$

18 $$\begin{aligned} \frac{\mathrm{d}\Theta _{i} }{\mathrm{d} t}= &{} C_{3} \Theta _{b}+ C_{4} \Theta _{i} \end{aligned}$$

### Steady state condition in network propagation

In large time limit, system will reach steady state. Rate of change of fractions *u*, *b* and *i* will be zero. In case of degree based compartment scheme, $u_{k}$, $b_{k}$ and $i_{k}$ will not change. Equating first and third equation of Eq. 7 to zero, we have

19 $$\begin{aligned} u_{k}= \frac{\mu }{\mu +\rho _{n} k \Theta _{b} } \; \quad{\text{and}}\; \quad i_{k}= \frac{\sigma b_{k}+(1-p)\rho _{n}ku_{k} \Theta _{b}}{\lambda +\mu +\alpha _{n} k p \Theta _{b}} \end{aligned}$$

Putting these values in second equation of the same set will give

20 $$\begin{aligned} b_{k}= \frac{\rho _{n} k \Theta _{b}(\mu p+\lambda +\alpha _{n} k p \Theta _{b})}{(\mu +\rho _{n} k \Theta _{b})(\lambda +\mu +\sigma +\alpha _{n} k p \Theta _{b})} \; \end{aligned}$$

Multiplying $b_{k}$ by $\frac{kp_{k}}{\left\langle k \right\rangle }$ and performing summation over *k*, we get

21 $$\begin{aligned} \Theta _{b}=\frac{1}{\left\langle k \right\rangle } \sum _{k} \frac{p_{k} k^{2} \rho _{n} \Theta _{b}(\mu p+\lambda +\alpha _{n} k p\Theta _{b})}{(\mu +\rho _{n} k \Theta _{b})(\lambda +\mu +\sigma +\alpha _{n}k p \Theta _{b})} \; \end{aligned}$$

This above expression is a self consistency equation of $\Theta _{b}$, i.e. Eq. 21 can be written as $\Theta _{b}=f(\Theta _{b})$.

### Simulation over networks

The simulations are performed over random network and scale-free network having 1024 nodes and average degree 10. Our random network follows Erdös-Rényi model with binomial degree distribution which converges to Poisson distribution if the network has very large number of nodes. The scale-free network that we have used for our analyses is generated using Barabási-Albert model of preferential attachment^57^ and it follows power law degree distribution having power exponent 3. Though, both random network and scale-free network are popular in network science, most of the real world networks do not follow the characteristics of any particular network model. Thus, we have simulated the flow of recommendation campaigns under different conditions over several real world networks. These analyses help us to understand the behavior in real social interaction scenarios. For our simulation studies over real networks, we collect some popular social networks from KONECT database^35^, namely, Hamster network, Email network and Jazz network. But, as these networks are not generated from any recommendation campaign, we have also generated a recommendation network from a referral experiment.

### Experiment for generation of recommendation network

We have selected 5 people who know each other and asked them to forward a mail with a make-believe referral marketing message. The policy for the recommendation is as follows: after forwarding the message to his contacts, a person will get credit points depending on the number of forwards his/her contacts made. For example, if Alice forwards the message to 10 friends, and out of 10, 4 persons forward the message further to some other people, then Alice wins 4 credit points. To track the propagation of the message and to the network, we have asked everyone to send one copy of the forwarded message to our groups’ email address, so that we can draw the connections between the participants. Two friends of Alice may send the message to each other even if both of them receive the message from Alice. Though, in our experiment, the network structure becomes a directed graph, but we assume that the network structure is undirected, because if ‘A’ can send a viral message to ‘B’, ‘B’ may also send some other viral message to ‘A’. As the experiment goes on, we also receive some isolated points and branches. The isolated branches may arise if someone forwards the message to other people but forgets to include our email id while forwarding the message, and his/her contacts forward the message following the instructions correctly. For the ease of discussion, we have removed the isolated branches while building the final network. Finally, we have a sparse network with 1,157 nodes and 2,558 edges.

## Data Availability

All data are available on request.
